# Prevalence, Perception and Predictors of Concomitant Herbal Medicine Use among HIV/AIDS and Tuberculosis Patients in Metekel Zone, Northwest Ethiopia: A Cross-Sectional Study

**DOI:** 10.1155/2022/8235229

**Published:** 2022-11-17

**Authors:** Mamo Feyissa, Teferi Gedif Fenta, Kaleab Asres, Tsige Gebremariam

**Affiliations:** ^1^Department of Pharmacology and Clinical Pharmacy, School of Pharmacy, College of Health Sciences, Addis Ababa University, Addis Ababa, Ethiopia; ^2^Department of Pharmaceutics and Social Pharmacy, School of Pharmacy, College of Health Sciences, Addis Ababa University, Addis Ababa, Ethiopia; ^3^Department of Pharmaceutical Chemistry and Pharmacognosy, School of Pharmacy, College of Health Sciences, Addis Ababa University, Addis Ababa, Ethiopia

## Abstract

**Background:**

The use of herbal medicine is common in Ethiopia. However, evidence on the extent and predictors of concomitant use of herbal medicine with conventional treatment among HIV/AIDS and tuberculosis patients is limited.

**Objective:**

To assess the extent of concomitant use of herbal medicine with conventional therapy and associated factors among HIV/AIDS and tuberculosis patients in Metekel Zone, Northwest Ethiopia.

**Method:**

A cross-sectional study was conducted from January to March 2020. HIV/AIDS and tuberculosis patients who visited the health facilities during the study were interviewed face-to-face using a structured and pretested questionnaire. The descriptive statistics and univariate and multivariate logistic regression analyses were conducted using SPSS version 25. A *P*-value of <0.05 was considered significant.

**Results:**

412 patients on conventional treatment were included in this study; 355 (86.2%) were HIV patients, and 57 (13.8%) were TB patients. More than half, 217 (52.7%) participants reported using herbal medicine while on conventional therapy. Among those who claimed to have used herbal medicines, 32 (14.7%) received herbal medicine from traditional healers. About four of five herbal users did not disclose their use to their healthcare providers. The type of health facility on follow-up (*P*=0.03), disease status (*P*=0.01), occupation (*P*=0.02), discontinuing ART (*P*=0.03), and encountering side (*P*=0.04) were the determinant factors for the use of herbal medicine among our study participants.

**Conclusion:**

In the Metekel Zone, concomitant consumption of herbal medication is common among HIV/AIDS and tuberculosis patients. Furthermore, most patients did not disclose the healthcare practitioners about their herbal use. Therefore, healthcare practitioners must assess and counsel patients regarding the potential adverse effects and herb-drug interaction to optimize therapy.

## 1. Introduction

The human immunodeficiency virus (HIV) remains a global challenge, particularly in sub-Saharan Africa, as it affects the young and economically productive population [1, 2]. An estimated 38.4 million people were living with HIV, with 1.5 million new infections in 2021. The Eastern and Southern Africa region carries more than half (54%) of the global HIV burden [1]. Ethiopia is among the most highly affected countries, with a 0.9% national HIV prevalence. Also, HIV/AIDS is among Ethiopia's top ten killer diseases [1, 3].

Human immunodeficiency virus/acquired immunodeficiency syndrome (HIV/AIDS) and tuberculosis (TB) are highly interrelated infectious diseases. TB is the leading infectious killer resulting in 1.6 million deaths yearly, of which 300,000 deaths were among people living with HIV/AIDS (PLWHA). TB account for one in three AIDS-related deaths [4]. On the other hand, HIV infection increases the risk of developing active TB by 16–27 times greater in PLWHA [5]. Ethiopia's TB/HIV coinfection prevalence is around 25.6% [6].

There is a widespread use of traditional medicine among PLWHA, although early initiation of antiretroviral therapy (ART) has reduced mortality and morbidity and improved their quality of life [7]. Several comorbid physical and psychosocial issues such as pain, opportunistic infections, side effects of ART, depression, and anxiety encountered by PLWHA contribute to the greater use of traditional medicine [8]. The chronic nature and incurability of HIV led patients to practice both conventional and traditional therapies to improve their quality of life and longevity. In Ethiopia, herbal medicine is the most commonly used traditional medicine [9].

The concomitant use of herbal medicine with ART has been reported to be 50%–95% [8, 10, 11]. This rate of herbal medicine use among PLWHA is about three times higher than among uninfected people [12]. PLWHA uses herbal medicines to manage ART side effects and opportunistic infections, meet primary healthcare needs, and treat HIV [8, 13–16]. Besides, herbal medicines are used for general well-being, relaxation, pain, stress, spiritualism, healing, improving energy level, and as a dietary supplement by PLWHA [16–18].

Many independent factors predict herbal medicines use among PLWHA. Several studies reported that higher herbal medicine use is associated with shorter duration since diagnosis (<5 years), shorter duration on ART (<4 years), experiencing opportunistic infections (OIs), the occurrence of ART side effects, living in a rural province, female gender, older age, being unmarried, employed, and limited education among HIV patients on ART [14, 15, 19, 20].

Even though herbal medicine use is common among PLWHA on ART, it is difficult to accurately determine the prevalence of concomitant use since patients rarely inform herbal use health care providers [15, 21]. According to studies, more than half of patients who used herbal medicine did not reveal their usage to healthcare [10, 18]. Similarly, Onifade et al. [22] reported that 73.4% of herbal medicine users denied use when asked by a medical practitioner.

Many patients perceive herbal remedies as effective and safe for managing HIV. A study revealed that 67.2% of the patients believe that herbal therapy is effective, and 64.1% use herbal therapy as complementary therapy [22]. This contributed to the continued use of herbal remedies concurrently with ART.

In Ethiopia, herbal medicine is widely used by HIV/AIDS patients [9]. However, there is limited evidence on the extent and predictors of herbal medicine use among PLWHA while on ART. Two studies conducted in Gondar Teaching Hospital, Northwest Ethiopia reported that 43.7% and 70.8% of patients use traditional medicines while on ART [10, 23]. A study in four referral hospitals revealed 28.5% concomitant herbal use among PLWHA [24]. The most common herbal preparations used by PLWHA were *Allium sativum*, *Moringa stenopetala*, and *Zingiber officinale* [10, 24].

Few studies assessed the Prevalence of herbal medicine use among TB patients. A study in Peru reported 63% of herbal use among TB patients [25]. However, herbal medicines are used by TB patients for symptomatic treatment [26], managing hepatotoxicity due to anti-TB drugs [27], prevention of TB resistance [28], and for their antimycobacterial activity [29].

The potential of herbal medicines to interfere with ART or anti-TB treatment effectiveness is a pressing concern due to little evidence supporting their safety and efficacy [30]. Indeed, using certain types of herbs may compromise the efficacy of ART due to an unanticipated drug interaction or side effects from herbs [31]. The potential for adverse outcomes may be amplified when patients do not disclose herbal medicine use to their healthcare providers and when the patient preference for herbs interferes with the uptake of conventional ART [10]. Hence, research determining the extent and the determinant factors for using herbal medicine is paramount to recognizing the impact of herbal use on HIV/AIDS and TB care. It also signals health care providers to consider herbal use during patient assessment and care and identify patients who need counseling on herbal use. In Ethiopia, no study has determined the extent of herbal medicine use among PLWHA receiving ART in primary healthcare facilities such as health centers. Therefore, this study aimed to assess the prevalence and contributing factors for use of herbal medicine among PLWHA and TB patients attending treatment in the health facilities of Metekel Zone, Northwest Ethiopia.

## 2. Methodology

### 2.1. Study Area

This research was carried out in the Metekel Zone, a rural area in the Benishangul Gumuz regional state, about 650 kilometers northwest of Addis Ababa. Recently, the Metekel zone has been a hot spot for spreading HIV and TB infections as small towns are booming due to significant government projects, including the Ethiopian great renaissance dam in the zone. Moreover, communities of the Metekel Zone are known for their culture of using herbal medicine for the management of different illnesses [32, 33].

This study was conducted in five health facilities in five different districts in Metekel Zone. The selected health facilities were Pawi General Hospital in the Pawi district, Gilgel Beles Heath Center in the Mandura district, Dibate Health Center in the Dibate district, Bullen Health Center in the Bullen district, and Debrezeit Health Center in the Wonbera district. Health facilities were selected purposively for this study based on the availability of both HIV/AIDS and TB care and treatment services in these facilities. Approximately 2000 HIV/AIDS patients were on active ART follow-up during the study period in the selected facilities. As an ambulatory care service, HIV/AIDS prevention, care, and treatment are provided in dedicated HIV clinics in each health facility. Similarly, the TB clinics offer care for patients in the intensive phase with directly observed therapy and continuation phase TB treatment as ambulatory care in each facility.

### 2.2. Study Design

A cross-sectional study using structured questionnaires was conducted from January 15 to March 15, 2020.

### 2.3. Source and Study Population

The source population consisted of all PLWHA and TB patients currently receiving conventional care and treatment in the Metekel Zone. In contrast, the study populations were all PLWHA and TB patients on conventional therapy, over 18, who visited ART and TB clinics for a refill during the study period and were willing to participate in the study.

### 2.4. Sample Size and Sampling

The sample size for PLWHA was calculated using single population proportion formula with the following assumptions: 95% confidence interval, 5% margin of error, and 50% prevalence of herbal use by PLWHA on ART as follows:

(1) $$\begin{array}{c} N={\left(\frac{z\alpha }{2}\right)}^{2}P\frac{\left(1-P\right)}{d^{2}},\\ n=\frac{1.96^{2}*0.5\left(1-0.5\right)}{0.05^{2}}=384. \end{array}$$

Since, the total this population is <10,000, Cochran's sample size correction formula for finite population size was applied.

(2) $$\begin{array}{c} nf=\frac{n0}{1+\left(n0-1\right)/N}=\frac{384}{1+\left(384-1\right)/2000}=323, \end{array}$$

where *nf* is the final corrected sample size, *n*_0_ is the sample size for an infinite population, and *N* is the population size.

Considering the 10% contingency for nonresponse, a total sample of 355 HIV/AIDS patients was included in the study. The samples were distributed proportionally to each study facility based on the total number of patients. Accordingly, 210 participants in Pawi Hospital and about 50 patients in each health center (Bullen, Dibate, Wonbera, and Mandura Health centers) were included in this study.

The total number of TB patients treated in the selected health facilities was small; therefore, all TB patients on follow-up during the study period were included. A convenient sampling technique was used where participants who voluntarily gave verbal consent were interviewed until the final sample size was reached for PLWHA.

### 2.5. Study Variables

The dependent variable was the concomitant use of herbal medicine and conventional treatment, whereas independent variables include sociodemographic characteristics of participants such as age, gender, religion, marital status, educational status, and occupation; and clinical characteristics of participants such as duration since diagnosis, duration on treatment, ART regimen, phase of treatment for TB, recent CD4 count, the experience of opportunistic infection, presence of comorbidity, and occurrence of ART side effects.

### 2.6. Data Collection Methods

Data were collected by six data collectors (2 data collectors in Pawi General Hospital and 1 data collector in each health center) selected among healthcare providers working in the same facilities as health officers or nurses. Data collectors were trained for a day by the research team on how to approach participants, interview them, and adequately fill in the required data.

Data were collected using a structured and pretested questionnaire, which was first prepared in English and then translated into the Amharic language (the official language of the study area). The survey questionnaire contained three sections; the sociodemographic characteristics (age, gender, marital status, occupation, and educational level); clinical characteristics (duration since diagnosis and ART initiation, current use of ART, CD4 count, and symptoms experienced and any side effect encountered during treatment); and the practice of herbal medicine use (commonly used herbs, reasons for use, and disclosure to healthcare providers). To maintain the quality of data, the questionnaire was pretested, and ambiguous statements were rephrased to improve clarity. The research team checked the completeness and accuracy of the data collected throughout the data collection process.

### 2.7. Data Analysis

The data were entered and analyzed using SPSS version 25. Descriptive statistics such as frequency distribution and percentages were determined. The independent association between herbal medicine use and sociodemographic and clinical characteristics was analyzed using univariate logistic regression. The variables with *P* < 0.25 in this model were kept for multivariate logistic regression to identify independent predictors for the concomitant use of herbal medicine adjusted for other factors. The *P*-value of <0.05 was considered significant.

#### 2.7.1. Glossary of Terms

- Traditional medicine is the total of the knowledge, skill, and practices based on the theories, beliefs, and experiences indigenous to different cultures, whether explicable or not, used in the maintenance of health as well as in the prevention, diagnosis, improvement, or treatment of physical and mental illness
- Herbal medicines include herbs, herbal materials, herbal preparations, and finished herbal products that contain active ingredients, parts of plants, other plant materials, or combinations.
- Traditional medical practitioner or traditional healer (TH) is a person who is recognized by the community where he lives as competent to provide health care by using vegetable, animal, mineral substances, and certain other methods.
- Concomitant herbal use: using herbal medicine for self-medication or using herbal therapies obtained from traditional medical practitioners while on conventional treatment.
- Self-medication is the selection and use of medicines (herbal and traditional products) by individuals to treat self-recognized illnesses or symptoms without consulting health care providers.

## 3. Results

### 3.1. Sociodemographic Characteristics of Study Participants

In this study, a total of 412 participants were included. About half of the participants were from Pawi General Hospital 210 (51%) and in the age group between 30–45 years 203 (49.3%) with a mean age of 37.1 (±10.4 SD). The majority of the study participants were females, 230 (55.8%), married 277 (67.2%), and followers of Orthodox Christianity 319 (77.4%). Regarding literacy status, about half of the participants had no formal education 194 (47.1%), and participants had primary school education 100 (24.3%). The details of sociodemographic characteristics are described in Table 1.

### 3.2. Clinical Characteristics of Participants

Out of the total participants, most were HIV patients on ART 355 (86.2%), and the remaining were TB patients 57 (13.8%). Most HIV patients were on ART for ≤5 years 148 (41.7%), on tenofovir/lamivudine/dolutegravir (TDF/3TC/DTG) regimen 193 (54.4%) and with 350+ CD4 count 269 (75.8%). Regarding TB patients, most were in the continuation phase of the treatment 36 (63.2%). Tables 2 and 3 describe the detailed clinical characteristics of the study participants.

### 3.3. Prevalence of Herbal Medicine Use, Reasons for Use, and Source of Information

**Figure 1:**
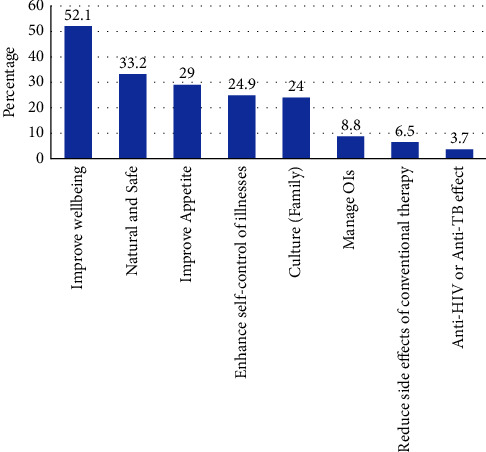
Reasons for using herbs among HIV/AIDS and TB patients in Mekel zone, northwest Ethiopia (OIs: opportunistic infections).

A total of 217 (52.7%) study participants used herbal medicine concomitantly with conventional therapy. The concomitant use of herbal medicine is significantly higher among the PLWHA than among TB patients (54.9% vs. 38.6%, OR = 1.94, *P*=0.012). The primary reasons for the use of herbal medicine by respondents were to improve general well-being 112 (52.1%), the belief that herbal medicine is natural and safe 72 (33.2%) and to improve appetite 63 (29.0%) (Figure 1).

As shown in Table 4, the most common herbal medicines used by HIV and TB patients were garlic (*Allium sativum*) 124 (57.4%) followed by Ginger (*Zingiber officinale*) 108 (50%), rue (*Ruta chalapensis*) 40 (18.5%), and moringa (*Moringa stenopetala*) 34 (15.3%). Regarding ethnopharmaceutical processing for herbal products, participants mostly prepared herbal remedies as decoctions and drank while hot as tea, chewed and swallowed, and infusions in hot tea/coffee. Study participants used herbal medicine for several indications, mostly for managing the common cold, sore throat, rash, herpes zoster, hepatitis, arthritis, poor appetite, and improving general well-being.

The major sources of information mentioned by herbal medicine users were family members 146 (67.2%), friends/neighbors 130 (59.9%), media platforms (social media, mass media, or internet) 22 (10.1%), and traditional healers 8 (3.7%).

### 3.4. Reasons for Not Using Herbal Medicine

**Figure 2:**
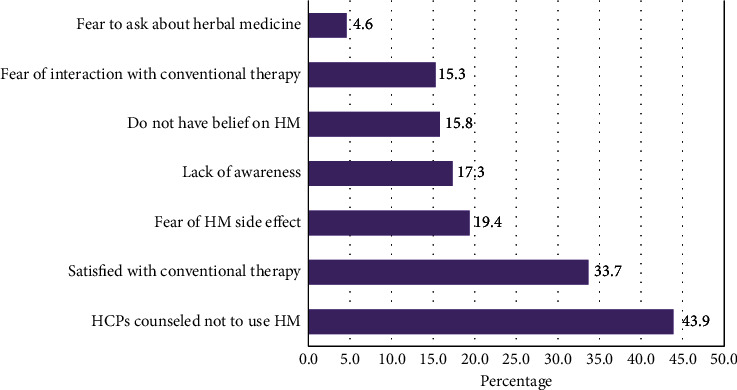
Reasons for not using herbal medicine among HIV/AIDS and TB patients, Metekel zone, northwest Ethiopia (HM: herbal medicine, HCPs: health care providers).

A total of 195 (47.6%) study participants said they did not use herbal medicine while on their conventional therapy ART or TB treatment. The reasons given by participants for not using herbal medicine were counseling of health care providers against the concomitant use of herbal medicine 88 (43.9%), being satisfied with conventional therapy 67 (33.7%), and fear of side effects of herbal remedies 38 (19.4%) (Figure 2).

### 3.5. Perception of Concomitant Use of Herbal Medicine

Most participants believe that herbal remedies effectively manage HIV/AIDS-related illnesses 376 (91.3%) but only 34 (8.3%) believe that herbal medicine can cure HIV/AIDS and/or TB. Furthermore, most participants believed herbal medicines were natural and safe 336 (81.6%) and did not interact with ART or anti-TB medications 365 (88.6%). Following an HIV or tuberculosis diagnosis, the majority of patients 369 (89.6%) sought first medical care from biomedical healthcare facilities. As the first action for the treatment of HIV/AIDS or TB, only 8 (1.9%) of the patients used herbal medicine while 39 (9.5%) used a religious practice like “Tsebel” (Table 5).

### 3.6. Communication with Health Care Providers about Herbal Medicine Use

Among claimed herbal medicine users, only 39 (18.0%) of the participants consulted traditional healers, and 32 (14.7%) received herbal medicine used by traditional healers. Most 173 (79.7%) concomitant herbal medicine users did not disclose herbal use to health care providers (HCPs). The cited reasons for nondisclosure were not being asked by HCPs 131 (75.7%), fear of discussing herbal use 40 (23.1%), and did not need the opinion of HCPs on herbal use 12 (6.9%). On the other hand, patients who disclosed their herbal medicine said HCPs were supportive and encouraged their use 34 (77.3%), and only five patients (11.4%) claimed to have received advice against herbal use.

### 3.7. Factors Associated with the Use of Herbal Medicine

The independent association between all sociodemographic and clinical variables with the use of herbal medicine was determined by Pearson's chi-square test. The sociodemographic factors such as age, gender, marital status, religion, and ethnicity were not significantly associated with herbal medicine use among our study participants. The odds of using herbal medicine are twice among HIV patients than among TB patients [AOR = 1.96 (1.02, 3.85), *P*=0.01]. Occupation of patients was a significant predictor of herbal medicine use where housewives (AOR = 1.74, *P*=0.03) and self-employed (AOR = 3.34, *P*=0.04) had higher odds of using herbal medicine than farmers. Among PLWHA, encountering side effects from ART [2.56 (1.05, 7.14), *P*=0.04] and discontinuing ART [2.05 (1.08, 3.85), *P*=0.03] were found to be determinants of herbal medicine use.  Table 6 shows the factors associated with herbal medicine use among the study participants.

## 4. Discussion

Despite the improved accessibility and early initiation of ART, there is still widespread use of herbal medicine among HIV patients. In this study, over half of the participants, 52.7% (54.9% PLWHA and 38.6% TB patients) used herbal medicine while on conventional treatment. Our finding on the herbal medicine use among PLWHA is comparable with reports from other African countries that reported a prevalence between 53.7% and 58% [11, 14, 34]. However, it is lower than the findings reported by Haile et al. [10] in northwest Ethiopia (70.8%) and another study in Lesotho (69.9%) [35] but higher than the prevalence of herbal medicine use reports by two studies in Uganda [15, 36]. The findings between studies might be due to differences in study populations, unstandardized definitions for herbal medicine, and inclusion/exclusion criteria for specific types of therapies in these studies.

Similarly, among TB patients included in the present study, 38.6% reported concomitant herbal medicine use. Generally, studies on the prevalence of herbal use among TB patients are limited. However, a study in Peru showed a higher rate than what was found in our study [25]. Besides, ethnobotanical studies indicated herbal medicines are used for the symptomatic treatment of TB [26], management of hepatotoxicity due to anti-TB drugs [27], prevention of TB resistance [28], and their antimycobacterial activity [29].

Improving general well-being, the perception that herbal medicines are natural and safe and improving appetite were the major reasons for using herbal medicine among our study participants. Similarly, a study by Littlewood and Vanable [37] reported alleviating HIV symptoms and ART side-effects and improving the quality of life as reasons for herbal medicine use. According to Lubinga et al. [36], the main reason for using herbal medicine is to treat symptoms related to OIs. In contrast, dissatisfaction with conventional therapy, 39.6% and belief in the advantages of herbal medicines was cited as primary reasons for use in another study done in Northwest Ethiopia [10]. The differences in perception, culture, and belief of the study population towards herbal medicines and ART would determine the reason for using herbal medicine by HIV/AIDS patients.

The most commonly used herbal remedies by our study participants were *A. Sativum*, 57.1% and *Z. officinale*, 49.7%. Similar to our finding, Z. *officinale,* and *A. sativum* have been reported as the most commonly used herbal remedies [10, 38]. Another study, however, stated *N. sativa* and *M. oleifera* as frequently used herbal products along with ART [23]. Despite the notion that herbal remedies are safe concurrent use with ARV drugs may lead to herb-drug interactions that may interfere with the effectiveness of ART, causing either potentially dangerous side effects and/or reduced benefits [15, 39]. Even the likelihood of herb-drug interactions could be higher than drug-drug interactions since herbs contain mixtures of pharmacologically active constituents [40–42]. There are proven adverse interactions between herbal medications and ARV drugs, African potato (*Hypoxis hemerocallidea*) can potentially inhibit ARV drug metabolism and transport, and garlic supplements have detrimental effects on the plasma concentrations of saquinavir [11]. Also, Cordova et al. [43] reported 2 cases of virologic breakthrough failure due to possible herb-drug interaction between horsetail *(Equisetum arvense)* and antiretroviral therapy.

Disclosing the HCPs about herbal therapies can help reduce the risk of harmful interactions by improving effective management, counseling, and treatment monitoring [16]. However, the disclosure of herbal medicine among patients in the present study is very low; only one out of five patients informed their HCPs that they use herbs. This finding is in line with the 15% disclosure rate by Issa et al. [24], but it is higher than the 7.7% disclosure rate in Uganda [36] and lower than 38.8% in Gondar, Ethiopia [10]. Patients preferred not to disclose herbal medicine use to HCPs due to fear of lack of support and understanding by HCPs [44]. Most patients think HCPs have a dismissive attitude toward traditional medicine because of the lack of credible evidence [45]. However, our study revealed that most HCPs (77%) are supportive and encourage patients who disclose their concomitant herbal use. However, considering the potential adverse herb-drug interaction and lack of evidence on the safety of herbal therapy, it is not appropriate to encourage patients to continue the use of herbal therapy. Therefore, HCPs need to inform patients of the potential for adverse herb-drug interaction and adverse outcomes.

Many patients believe herbal medicines are natural, safe, and culturally acceptable remedies to treat several illnesses. In the present study, 91.3% of participants believe herbal remedies are effective and 81.6% think herbal medicines are natural and safe with no adverse effects. Besides, only 11.4% of participants were aware of the potential interaction with conventional medicine. This finding is consistent with the study in Nigeria, where 34.7% of HIV patients believed that herbal remedies are natural [34]. Using relatively small quantities of herbs and spices as part of a regular diet is not likely to cause adverse herb-drug interactions, but using a higher amount for medicinal purposes might increase the likelihood of adverse interaction with conventional therapy [46]. For instance, adverse interactions are documented with the medicinal use of *garlic* with anticoagulants, antihypertensives, antiviral, anticancer, antidiabetic, and antitubercular drugs [46–48]. The interactions between garlic and antiretroviral protease inhibitors may lead to a decreased concentration of protease inhibitors and increased resistance to ART [31].

Most HIV/AIDS and TB patients in our study practiced self-medication of herbal medicine as a homemade remedy for self-care. The patients used information from families, friends, and diverse sources to choose herbal medicine. Among our participants, 14.7% received herbal medicine prescribed by healers. This is consistent with a report where 18.8% of PLWHA used herbs prescribed by traditional healers in Gondar, Ethiopia [23]. Despite having little evidence about their role, many traditional healers are involved in treating HIV/AIDS in Africa [49]. However, the role of traditional healers in managing HIV/AIDS remained unexplored in Ethiopia.

Several factors independently predict the use of herbal medicine among patients. In the present study, significant predictors of herbal medicine use were the type of healthcare facility on follow-up (*P* ≤ 0.03), disease state [HIV vs. TB] (*P*=0.02), occupation (*P*=0.01), encountering side effects from ART (*P*=0.04), and history of discontinuing ART (*P*=0.03). Our finding is similar to the reports that independent predictors of herbal medicine use among HIV/AIDS patients are a history of experiencing side effects from ART [15, 20, 36], nonadherence to ART (15), and occupation [14]. Despite our findings, several other factors such as older age, female sex [20], the occurrence of OIs [20, 37], educational level [10, 11], duration on ART, self-perceived health status [36], and perceptions towards herbal therapy [12] are found to be independent predictors for herbal medicine among HIV patients.

### 4.1. Strengths and Limitations of the Study

The strength of this study involves multiple health facilities such as a hospital and health centers; hence, the finding can well represent the extent of herbal medicine use among HIV and TB patients in the Metekel zone. Furthermore, the selected study site is an unexplored rural area where a majority of the community rely on traditional medicine for primary health care. However, this finding cannot be extrapolated to general concomitant herbal use among PLWHA and TB while on conventional therapy. This study was based on a cross-sectional design; hence, causal inference between independent factors and herbal medicine use cannot be made. Moreover, as the participants were asked about their past use of herbal medicine, recall bias may contribute to underreporting of the use.

## 5. Conclusion and Recommendations

The concomitant use of herbal medicines with conventional ART and anti-TB drugs is high among PLWHA and TB patients in the Metekel zone. The most commonly consumed herbal remedies were *A. sativum* and *Z. officinale* as homemade remedies. The main reasons for utilizing herbal medicine were the improvement of general well-being and the belief that herbal therapy is natural and safe. Besides, the majority of participants who utilized herbal medicine did not inform their healthcare providers. Therefore, healthcare providers must proactively assess and advise patients' concomitant use of herbal medicine.

On the other hand, most HCPs in the Metekel zone encouraged the use of herbal medicine for those who disclosed their use despite a lack of supporting evidence. The counseling on herbal medicine should address the benefits, potential adverse herb-drug interaction, and toxicities of concomitant use with ART or anti-TB drugs. Training is required for HCPs to appropriately counsel patients on herbal medicine use. Therefore, the Federal Ministry of Health should develop a guideline for HCPs to assess and counsel patients on herbal medicine use effectively. In addition, training health care providers on herbal medicine are also paramount to improve the safe use of herbal medicine.

## Figures and Tables

**Table 1:** Sociodemographic characteristics of study participants, Metekel zone, northwest Ethiopia (*N* = 412).

Characteristics		Frequency	Percent
Health facility	Pawi general hospital	210	51.0
	Bullen HC	50	12.1
	Dibate HC	51	12.4
	Gilgel beles HC	52	12.6
	Debrezeit HC	49	11.9

Clinic	ART clinic	355	86.2
	TB clinic	57	13.8

Gender	Male	182	44.2
	Female	230	55.8

Age	≤30	130	31.5
	30–40	163	39.6
	40–50	82	19.9
	51+	37	9.0

Marital status	Single	58	14.1
	Married	277	67.2
	Widow	36	8.7
	Divorced	41	10

Religion	Orthodox	319	77.4
	Muslim	69	16.7
	Protestant	24	5.8

Educational status	No formal education	194	47.1
	Primary school	100	24.3
	Secondary school	73	17.7
	Certificate/diploma	30	7.3
	Degree and above	15	3.6

Occupation	Farmer	141	34.2
	Housewife	99	24.0
	Public servant	58	14.1
	Self-employee	46	11.2
	Student	30	7.3
	Others^*∗*^	38	9.2

HC, health center; ART, antiretroviral therapy; TB, tuberculosis; ^*∗*^others include daily laborers, jobless, and drivers.

**Table 2:** **C**linical characteristics of HIV/AIDS patient study participants, Metekel zone, northwest Ethiopia (*N* = 355).

Variable		Frequency (%)
Know HIV status	≤5 years	141 (39.7)
	5–10 years	131 (36.9)
	10+ years	83 (23.4)
ART duration	≤5 years	148 (41.7)
	5–10 years	138 (38.9)
	10+ years	69 (19.4)
ART regimens	TDF/3TC/DTG	193 (54.4)
	TDF/3TC/EFV	136 (38.3)
	AZT/3TC/NVP	6 (1.7)
	AZT/3TC/ATV/r	20 (5.6)
Current CD4 count	≤350	86 (24.2)
	350+	269 (75.8)
Comorbidity	Yes	70 (19.7)
	No	285 (80.3)
Use of other medication	Yes	108 (30.4)
	No	247 (69.6)
Experiencing OIs	Yes	66 (18.6)
	No	289 (81.4)
Developed ART side effects	Yes	22 (6.2)
	No	333 (93.8)
History of drug discontinuation	Yes	54 (15.2)
	No	30 (14.8)

HIV: human immunodeficiency virus, TB: tuberculosis, ART: antiretroviral therapy, TDF: tenofovir disoproxil fumarate, 3TC: lamivudine, DTG: dolutegravir, AZT: zidovudine, EFV: efavirenz, and NVP: nevirapine.

**Table 3:** Clinical characteristics of TB study participants, Metekel zone, northwest Ethiopia (*N* = 57).

Variable		Frequency *n* (%)
Phase of TB treatment	Intensive phase	21 (36.8)
	Continuation phase	36 (63.2)

Comorbidity	Yes	3 (5.3)
	No	54 (94.7)

Develop side effect	Yes	1 (1.8)
	No	56 (98.2)

Use of other medication	Yes	3 (5.3)
	No	54 (94.7)

**Table 4:** Herbal remedies used by HIV and TB patients while on conventional treatment, methods of preparation, and parts used, in Metekel zone, northwest Ethiopia.

Scientific name	Common name	Frequency *n*(%)^*∗*^	Used for	Plant part used	Preparation and administration
*Allium sativum* L.	Nech-shinkurt (*A*) Garlic (*E*)	124 (57.41)	Cough, general well-being, common cold, sore throat to improve appetite, tonsillitis, swelling, gout (together with honey), and kurtimat (arthritis)	Bulb	The bulb is chewed and swallowed, crushed and boiled in water, and taken as a tea used with food
*Zingier officinale* roscoe	Zinjibil (*A*) Ginger (E)	108 (50.00)	Cough, general well-being, sore throat, to improve appetite, and the common cold	Rhizome	Rhizomes are chewed. Rhizomes are freshly pulverized and then boiled; the decoction is taken while warm
*Ruta chalapensis* L.	Tena'Adam (*A*) Rue (*E*)	40 (18.52)	Cough, common cold, and mich (rash)	Leaf	Infusion of the leaf in hot tea or coffee to be taken orally
*Moringa stenopetala* (baker f.) cufod	Shiferaw (*A*) Moringa (*E*)	34 (15.28)	Cough, common cold, several illnesses, well-being, asthma, and hypertension	Leaf seed	The fresh or dried moringa leaf or seed pounded prepared as a tea
*Nigella sativa* L.	Tikur-azmud (*A*) black cumin (*E*)	14 (6.48)	Mich (rash) and cough	Seed	The powdered seed is mixed with water

*Zheneria scabra* sond	Haregresa (*A*)	13 (6.02)	Mitch (rash)	Leaf	Fresh leaves are crushed and the volatile substance is inhaled nasally
			Breast swelling	Root	Boiling with water and administered orally

*Ocimum lamiifolium* hochst	Damakase (*A*)	13 (5.56)	Common cold, cough, and mitch	Leaf	Fresh leaves are milled, the juice is prepared and then taken orally
	Sedo (*A*)	7 (3.24)	Peptic ulcer disease and appetite	Leaf	Crushed and mixed with juices of cereals, then eaten with porridge or injera
	Unknown herb	6 (2.78)	Herpes zoster and hepatitis		Taken orally
*Capsicum annuum* L.	Karia (*A*)	5 (2.31)	Appetite and tonsillitis	Fruit	Chewed with food
*Eucalyptus globules* labill.	Bahirzaf (*A*) Eucalyptus (*E*)	3 (1.39)	Mitch (rash)	Leaf	The leaves are grounded and macerated in water, then four red hot stones are transferred into the mix and fumigated
*Apis mellifera* L.	Honey (*E*)	3 (1.39)	Arthritis and cold		Taken orally

*A*: Amharic, *E*: English; ^*∗*^reporting use of multiple herbal medicines were possible.

**Table 5:** The perception of HIV/AIDS and tuberculosis patients on herbal medicine use in Metekel zone, northwest Ethiopia (*n* = 412).

Question items	Yes (%)	No (%)
(1) Do you think herbal medicines are effective in the treatment of HIV/AIDS-related illness and TB	376 (91.3)	36 (8.7)
(2) Do you believe herbal medicines can cure HIV/AIDS or TB	34 (8.3)	378 (91.7)
(3) Do you think that herbal medicine interacts with your antiretroviral or antituberculosis medications	47 (11.4)	365 (88.6)
(4) Do you think herbal medicines are natural and safe	336 (81.6)	76 (18.4)
(5) Does concurrent use of herbal medicines cause adverse effects	40 (9.7)	372 (90.3)

**Table 6:** Determinants of herbal medicine use among people living with HIV/AIDS (PLWHA) and tuberculosis (TB) patients in Metekel zone, northwest Ethiopia (*n* = 412).

Characteristics		Use of HM, *n* (%)		Univariate analysis	Multivariate analysis	
		Yes	No	COR (95% CI)	AOR (95% CI)	*P* value
Type of facility	Pawi hospital	97 (46.2)	113 (53.8)	1	1	
	Health centers	120 (59.4)	82 (40.6)	1.71 (1.15, 2.52)	1.63 (1.04, 2.58)	0.03
Clinic	TB clinic	22 (38.6)	35 (61.4)	1	1	
	ART clinic	194 (54.6)	161 (45.4)	1.94 (1.09, 3.44)	1.96 (1.02, 3.85)	0.01
Gender	Male	89 (48.9)	93 (51.1)	1	1	
	Female	127 (55.2)	103 (44.8)	1.31 (0.89, 1.94)	1.04 (0.61, 1.75)	0.89
Age	≤30	63 (48.5)	67 (51.5)	1	1	0.16
	30–40	88 (54.0)	75 (46.0)	1.25 (0.79, 1.98)	1.24 (0.73, 2.11)	0.43
	41–50	50 (61.0)	32 (39.0)	1.66 (0.95, 2.91)	1.84 (0.95, 3.57)	0.07
	51+	16 (43.2)	21 (56.8)	0.81 (0.39, 3.69)	0.78 (0.35, 1.76)	0.55
Occupation	Farmer	64 (45.1)	78 (54.9)	1	1	0.02
	Housewife	62 (63.3)	36 (36.7)	2.11 (1.24, 3.57)	1.74 (1.02, 3.27)	0.03
	Public servant	32 (55.2)	26 (44.8)	1.48 (0.80, 2.74)	1.46 (0.77, 2.77)	0.25
	Self-employee	30 (81.8)	16 (18.2)	2.26 (1.13, 4.50)	4.86 (1.97, 24.4)	0.04
	Student	12 (40.0)	18 (60.0)	0.80 (0.36, 1.79)	0.90 (0.39, 1.78)	0.82
	Others^*∗∗*^	16 (42.1)	22 (57.9)	0.88 (0.42, 1.81)	0.82 (0.33, 1.13)	0.63
Current CD4 count^#^	≤350	51 (60)	34 (40)	1	1	
	350+	142 (52.6)	128 (47.4)	0.72 (0.42, 1.26)	0.73 (0.45, 1.21)	0.23
Occurrence of OIs^#^	No	153 (52.9)	136 (47.1)	1	1	
	Yes	40 (60.6)	26 (39.4)	1.37 (0.8, 2.36)	0.99 (0.55, 1.77)	0.9
Encountered SE from ART^#^	No	177 (53.2)	156 (46.8)	1	1	
	Yes	16 (72.7)	6 (27.3)	3.3 (1.09, 8.4)	2.56 (1.05, 7.14)	0.04
Ever discontinued ART^#^	No	155 (51.5)	146 (48.5)	1	1	
	Yes	38 (70.4)	16 (29.6)	2.24 (1.2, 4.19)	2.05 (1.08, 3.85)	0.03

HB: herbal medicine, COR: crude odds ratio, AOR: adjusted odds ratio, CI: confidence interval, ART: antiretroviral therapy, SE-ART: side effect, ART: antiretroviral therapy, OIs: opportunistic infection, ^#^variable applies only for PLWHA, ^*∗*^others: hyedya, gumuz, and kembata; ^*∗∗*^others: jobless and daily laborers.

## Data Availability

The datasets supporting the study's conclusions are available with the first author.

## Abbreviations

ART: Antiretroviral therapy
AIDS: Acquired immunodeficiency syndrome
AOR: Adjusted odds ratio
CI: Confidence interval
HIV: Human immunodeficiency virus
HCP: Health care providers
OI: Opportunistic infections
PLWHA: People living with HIV/AIDS
TB: Tuberculosis
WHO: World health organization
UNAIDS: United Nation agency for international development.

## References

[1] United Nations Publications (2022). *UNAIDS Global AIDS Update 2022*.

[2] Odugbesan J. A., Rjoub H. (2019). Relationship among HIV/AIDS prevalence, human capital, good governance, and sustainable development: empirical evidence from sub-saharan Africa. *Sustainability*.

[3] FMOH (2016). *Health and Health-Related Indicators 2008 EFY (2015/2016)*.

[4] WHO (2018). *HIV/TB*.

[5] Kwan C. K., Ernst J. D. (2011). HIV and tuberculosis: a deadly human syndemic. *Clinical Microbiology Reviews*.

[6] Tesfaye B., Alebel A., Gebrie A., Zegeye A., Tesema C., Kassie B. (2018). The twin epidemics: prevalence of TB/HIV co-infection and its associated factors in Ethiopia; a systematic review and meta-analysis. *PLoS One*.

[7] Chibawara T., Mbuagbaw L., Kitenge M., Nyasulu P. (2019). Effects of antiretroviral therapy in HIV- positive adults on new HIV infections among young women: a systematic review protocol. *Systematic Reviews*.

[8] Kelso-chichetto N. E., Okafor C. N., Harman J. S., Canidate S. S., Cook C. L., Cook R. L. (2016). Complementary and alternative medicine use for HIV management in the state of Florida: medical monitoring project. *Journal of Alternative & Complementary Medicine*.

[9] Kloos H., Mariam D. H., Kaba M., Tadele G. (2013). Traditional medicine and HIV/AIDS in Ethiopia: herbal medicine and faith healing: a review. *The Ethiopian Journal of Health Development*.

[10] Haile K. T., Ayele A. A., Mekuria A. B., Demeke C. A., Gebresillassie B. M., Erku D. A. (2017). Traditional herbal medicine use among people living with HIV/AIDS in Gondar, Ethiopia: do their health care providers know?. *Complementary Therapies in Medicine*.

[11] Langlois-klassen D., Jhangri G. S., Kipp W., Rubaale T. (2007). Use of traditional herbal medicine by AIDS patients in kabarole district, western Uganda. *The American Journal of Tropical Medicine and Hygiene*.

[12] Limsatchapanich S., Sillabutra J., Nicharojana L. O., Section C. P., Provincial S., Health P. (2013). Factors related to the use of complementary and alternative medicine among people living with HIV/AIDS in Bangkok, Thailand. *Health Science Journal*.

[13] Cichello S., Tegegne S. M., Yun H. (2014). Herbal medicine in the management and treatment of HIV-AIDS—a review of clinical trials. *Australian Journal of Herbal Medicine*.

[14] Gyasi R. M., Tagoe-Darko E., Mensah C. M. (2013). Use of traditional medicine by HIV/AIDS patients in kumasi metropolis, Ghana: a cross-sectional survey. *American International Journal of Contemporary Research*.

[15] Namuddu B., Kalyango J. N., Karamagi C. (2011). Prevalence and factors associated with traditional herbal medicine use among patients on highly active antiretroviral therapy in Uganda. *BMC Public Health*.

[16] Canadian AIDS Treatment Information Exchange (CATIE) (2005). *Practical Guide to Herbal Therapies for People Living with HIV*.

[17] Maroyi A. (2014). Alternative medicines for HIV/AIDS in resource-poor settings: insight from traditional medicines use in sub-saharan Africa. *Tropical Journal of Pharmaceutical Research*.

[18] Orisatoki R. O., Oguntibeju O. O. (2010). The role of herbal medicine use in HIV_AIDS treatment. *Insight Medical Publishing*.

[19] Hughes G. D., Puoane T. R., Clark B. L., Wondwossen T. L., Johnson Q., Folk W. (2012). Prevalence and predictors of traditional medicine utilization among persons living with aids (PLWA) on antiretroviral (ARV) and prophylaxis treatment in both rural and urban areas in South Africa. *African Journal of Traditional, Complementary and Alternative Medicines: AJTCAM*.

[20] Shiferaw A., Baye A. M., Amogne W., Feyissa M. (2020). Herbal medicine use and determinant factors among HIV/AIDS patients on antiretroviral therapy in tikur anbessa specialized hospital, Addis Ababa, Ethiopia. *HIV*.

[21] Liu J. P., Manheimer E., Yang M. (2005). Herbal medicines for treating HIV infection and AIDS. *Cochrane Database of Systematic Reviews*.

[22] Onifade A. A., Ajeigbe K. O., Omotosho I. O., Rahamon S. K., Oladeinde B. H. (2013). Attitude of HIV patients to herbal remedy for HIV infection in Nigeria. *Nigerian Journal of Physiological Sciences*.

[23] Endale Gurmu A., Teni F. S., Tadesse W. T. (2017). Pattern of traditional medicine utilization among HIV/AIDS patients on antiretroviral therapy at a university hospital in northwestern Ethiopia: a cross-sectional study. *Evidence-Based Complementary and Alternative Medicine*.

[24] Issa A., Messele B., Teshome D., Tilahun Z., Gedif T. (2017). Concomitant use of medicinal plants with antiretroviral drugs among HIV/AIDS patients in Ethiopia: a cross-sectional study. *European Physical Journal*.

[25] Oeser C. C., Evans C. A. W., Friedland J. S., Gilman R. H., Moore D. A. J., Escombe A. R. (2005). Does traditional medicine use hamper efforts at tuberculosis control in urban Peru?. *The American Journal of Tropical Medicine and Hygiene*.

[26] Moe S., Naing K. S., Htay M., Htay N. (2018). Herbal medicines used by tuberculosis patients in Myanmar. *European Journal of Medicinal Plants*.

[27] Liu Q., Garner P., Wang Y., Huang B., Smith H. (2008). Drugs and herbs given to prevent hepatotoxicity of tuberculosis therapy: systematic review of ingredients and evaluation studies. *BMC Public Health*.

[28] Pandit R., Singh P. K., Kumar V. (2015). Natural remedies against multi-drug resistant *Mycobacterium tuberculosis*. *Journal of Tuberculosis Research*.

[29] Gautam A. H., Ramica S., Rana A. C. (2012). Review on herbal plants useful in tuberculosis. *International Research Journal of Pharmacy*.

[30] Ekor M. (2014). The growing use of herbal medicines: issues relating to adverse reactions and challenges in monitoring safety. *Frontiers in Pharmacology*.

[31] Staines S. S. (2011). Herbal medicines: adverse effects and drug-herb interactions. *Journal of the Malta College of Pharmacy Practice*.

[32] Guji T., Gedif T., Gebre-Mariam T. (2013). Ethnopharmaceutical study of medicinal plants of Metekel zone, Benishangul-Gumuz regional state, mid-west Ethiopia. *Ethiopian Pharmaceutical Journal*.

[33] Genet A. (2018). Indigenous herbal medicinal knowledge among the shinasha. *Global Scientific Journals*.

[34] Idung A. U., Abasiubong F. (2014). Complementary and alternative medicine use among HIV-infected patient’s on anti-retroviral therapy in the Niger delta region, Nigeria. *Clinical Medicine Research*.

[35] Mugomeri E., Chatanga P., Chakane N. (2016). Medicinal herbs used by hiv-positive people in Lesotho. *African Journal of Traditional, Complementary and Alternative Medicines*.

[36] Lubinga S. J., Kintu A., Atuhaire J., Asiimwe S. (2012). Concomitant herbal medicine and antiretroviral therapy (ART) use among HIV patients in Western Uganda: a cross-sectional analysis of magnitude and patterns of use, associated factors and impact on ART adherence. *AIDS Care*.

[37] Littlewood R. A., Vanable P. A. (2008). Complementary and alternative medicine use among HIV-positive people: research synthesis and implications for HIV care. *AIDS Care*.

[38] Bahall M. (2017). Prevalence, patterns, and perceived value of complementary and alternative medicine among HIV patients: a descriptive study. *BMC Complementary and Alternative Medicine*.

[39] Yaheya M., Ismail M. (2009). Herb-drug interactions and patient counseling. *International Journal of Pharmacy and Pharmaceutical Sciences*.

[40] Fugh-Berman A., Ernst E. (2001). Herb-drug interactions: review and assessment of report reliability. *British Journal of Clinical Pharmacology*.

[41] Cordier W., Steenkamp V. (2011). Drug interactions in African herbal remedies. *Drug Metabolism and Drug Interactions*.

[42] Izzo A. A. (2012). Interactions between herbs and conventional drugs: overview of the clinical data. *Medical Principles and Practice*.

[43] Cordova E., Morganti L., Rodriguez C. (2017). Possible drug—herb interaction between herbal supplement containing horsetail (equisetum arvense) and antiretroviral drugs: report of 2 cases. *Journal of the International Association of Physicians in AIDS Care*.

[44] Puoance T., Hughes G., Uwimana J., Folk W. (2012). Why HIV positive patients on antiretroviral treatment and/or cotrimoxazole prophylaxis use traditional medicine: perceptions of health workers, traditional healers, and patients: a study in two provinces of South Africa. *African Journal of Traditional, Complementary and Alternative Medicines*.

[45] Pappas S., Perlman A. (2002). Complementary and alternative medicine: the importance of doctor-patient communication. *Medical Clinics of North America*.

[46] Izzo A. A., Ernst E. (2009). Interactions between herbal medicines and prescribed drugs an updated systematic review. *Drugs*.

[47] Adhikari A., Indu R., Sur T. K., Banerjee D., Kumar A. (2015). Is garlic a safe remedy: an overlook herb-drug interaction?. *AJPCT*.

[48] Gall A., Shenkute Z. (2009). *Ethiopian Traditional and Herbal Medications and Their Interactions with Conventional Drugs*.

[49] Audet C. M., Ngobeni S., Wagner R. G. (2017). Traditional healer treatment of HIV persists in the era of ART: a mixed-methods study from rural South Africa. *BMC Complementary and Alternative Medicine*.

